# Identification of key eRNAs for intervertebral disc degeneration by integrated multinomial bioinformatics analysis

**DOI:** 10.1186/s12891-024-07438-6

**Published:** 2024-05-04

**Authors:** Yongai Li, Runzhi Huang, Jianxin Ye, Xiaying Han, Tong Meng, Dianwen Song, Huabin Yin

**Affiliations:** 1 Department of Orthopedics, Shanghai General Hospital, Shanghai Jiao Tong University School of Medicine, Shanghai, China; 2 Department of Burn Surgery, The First Affiliated Hospital of Naval Medical University, Shanghai, China; 3 Department of General Surgery, The Second Affiliated Hospital, Zhejiang University School of Medicine, Hangzhou, China

**Keywords:** IVDD, eRNA, CTNNB1, Bioinformatics analysis

## Abstract

**Background:**

Intervertebral disc degeneration (IVDD) is a common degenerative condition leading to abnormal stress distribution under load, causing intervertebral stenosis, facet joint degeneration, and foraminal stenosis. Very little is known about the molecular mechanism of eRNAs in IVDD.

**Methods:**

Gene expression profiles of 38 annulus disc samples composed of 27 less degenerated discs (LDs) and 11 more degenerated discs (MDs) were retrieved from the GEO database. Then, differentially expressed enhancer RNAs (DEeRNAs), differentially expressed target genes (DETGs), and differentially expressed transcription factors (DETFs), hallmark of cancer signalling pathways according to GSVA; the types and quantity of immune cells according to CIBERSORT; and immune gene sets according to ssGSEA were analysed to construct an IVDD-related eRNA network. Then, multidimensional validation was performed to explore the interactions among DEeRNAs, DETFs and DEGs in space.

**Results:**

A total of 53 components, 14 DETGs, 15 DEeRNAs, 3 DETFs, 5 immune cells, 9 hallmarks, and 7 immune gene sets, were selected to construct the regulatory network. After validation by online multidimensional databases, 21 interactive DEeRNA-DEG-DETF axes related to IVDD exacerbation were identified, among which the C1S-CTNNB1-CHD4 axis was the most significant.

**Conclusion:**

Based upon the results of our study, we theorize that the C1S-CTNNB1-CHD4 axis plays a vital role in IVDD exacerbation. Specifically, C1S recruits CTNNB1 and upregulates the expression of CHD4 in IVDD, and subsequently, CHD4 suppresses glycolysis and activates oxidative phosphorylation, thus generating insoluble collagen fibre deposits and leading to the progression of IVDD. Overall, these DEeRNAs could comprise promising therapeutic targets for IVDD due to their high tissue specificity.

**Supplementary Information:**

The online version contains supplementary material available at 10.1186/s12891-024-07438-6.

## Introduction

Current therapies for intervertebral disc degeneration (IVDD) mainly include conservative and surgical treatments, which are supportive treatments but cannot cure IVDD [1, 2]. Investigations into biological regeneration treatments for IVDD have been increasing in recent years and have mainly focused on intervertebral disc regeneration [3, 4]. Nevertheless, because of the complex mechanisms of IVDD, these therapeutic techniques have not yet been widely utilized in the clinic. Hence, the identification of potential biomarkers that cause IVDD may help reveal the underlying mechanisms, providing novel targets for IVDD treatment.

Enhancer RNAs (eRNAs), long noncoding RNAs of 0.5-5 kb, are transcripts of highly histone-methylated enhancer regions [5]. Compared with general lncRNAs, eRNAs are mostly nonpolyadenylated and have a low stability, low abundance, short length, and short half-life; thus, eRNAs are considered nonfunctional transcriptional noise or transcriptional byproducts [6]. However, as cis-acting elements, eRNAs have been shown to recruit RNA polymerase II and transcription factors after being transcribed by enhancer units to regulate the expression of targeted or nearby genes [7, 8]. eRNAs, including HCG18 [9] (NR_024052.2), SNHG1 [10, 11] (NR_003098.2), H19 [12] (NR_002196.3), NEAT1 [13] (NR_028272.1), and linc-ADAMTS5 [14] (XR_007089974.1), were confirmed to play important roles in the occurrence and exacerbation of IVDD. Regulating the synthesis or degradation of the IVD extracellular matrix (ECM) is the most common method by which these eRNAs affect IVDD. For example, H19 and NEAT1 were upregulated in clinical IVDD specimens compared with normal IVD specimens. Both elevated H19 and NEAT1 promoted the expression of matrix metalloproteinases (MMPs) and ADAMTS5 (an aggrecan-degrading enzyme), while H19 could also elicit oxidative stress in nucleus pulposus (NP) cells, causing cellular senescence. Furthermore, linc-ADAMTS5 downregulation in IVDD led to the epigenetic silencing of ADAMTS5 via the recruitment of a transcription factor called RREB1 to the binding site overlapping the ADAMTS5 promoter. In addition to destabilizing the balance between ECM synthesis and degradation, inhibiting NP cell proliferation and promoting NP cell apoptosis are the mechanisms by which HCG18 and SNHG1 cause IVDD. However, these IVDD-related eRNAs were identified by lncRNA or mRNA microarrays, not systemic eRNA detection, and the functions of eRNAs have not been fully elucidated in IVDD. These findings indicated that eRNAs may play critical roles in IVDD; thus, these molecules could be utilized as biomarkers or therapeutic targets in clinical practice.

In this study, we constructed a regulatory network including 14 DETGs, 15 DEeRNAs, 3 DETFs, 5 immune cells, 9 hallmarks, and 7 immune gene sets. In addition, 21 interactive DEeRNA-DETF-DEG axes related to IVDD were identified. These DEeRNAs are promising therapeutic targets for IVDD due to their high tissue specificity.

## Materials and methods

### Data acquisition

The expression profiles and clinical information of 38 annulus disc samples consisting of 27 less degenerated discs (LDs) (Thompson grade I-III) and 11 more degenerated discs (MDs) (Thompson grade IV and V) samples were acquired from the Gene Expression Omnibus (GEO) database (http://www.ncbi.nlm.nih.gov/geoaccession numbers: GSE15227 [15] and GSE23130 [16]). These two sets of expression profiles were obtained via microarray analysis ([U133_X3P] Affymetrix Human X3P Array) and collected from the same platform, GPL1352. Expression profiles containing eRNAs and corresponding target genes in IVDD were extracted for subsequent analysis based on previous studies [17]. The expression profiles of immune-related genes and transcription factors (TFs) were retrieved from the ImmPort database [18] (http://www.immport.org/) and the Cistrome Cancer database [19] (http://cistrome.org/), respectively. ChIP-seq data for H3K27ac (accession numbers: GSM1195331 [20], GSM935653 [21] and GSM722419 [22]) and ATAC-seq data for eRNAs (accession number: GSE139099 [23]) were downloaded from the GEO database.

### Data processing and differentially expressed gene analysis

Patients with incomplete clinical information were excluded from this study. All original microarray data were analysed by using the affy package in R (https://bioconductor.org/biocLite.R). The robust multiarray average (RMA) algorithm was utilized to correct the background of the microarray data and normalize the microarray data. According to the Thompson grading criteria, Grade I and II samples were considered nondegenerative, while Grade III to V samples were considered IVDD. DEeRNAs, DEGs, and DETFs between LDs and MDs were identified using the Linear Models for Microarray Data (limma) package [24] under |log2FC (fold change)| > 1 and a false discovery rate (FDR) < 0.05.

### Functional enrichment analysis

Gene Ontology (GO) analysis was conducted by using the clusterProfiler R package, which revealed the functional enrichment of biology process (BP), cell component (CC), and molecular function (MF) terms. Reactome pathway analysis of eRNA-related coding genes was also conducted according to coexpression analysis. To avoid the accumulation of type I errors, enrichment items meeting FDR < 0.05 were considered significant. In addition, Kyoto Encyclopedia of Genes and Genomes (KEGG) analysis was performed using the clusterProfiler R package, and KEGG pathways with an FDR < 0.05 were considered significant.

### Immune cell infiltration analysis

The types and quantity of immune-infiltrating cells in LD and MD samples were estimated by cell type identification with the CIBERSORT algorithm [25] based on relative subsets of RNA transcripts. The immune-infiltrating cells with a CIBERSORT output of *P* < 0.05 were subsequently selected for nonparametric tests.

### GSVA and ssGSEA

Significant immune cells and immune-related signalling pathways were evaluated and selected by ssGSEA [26] from 29 immune-associated gene sets, including a total of 707 genes. Potential downstream hallmarks of cancer signalling pathways of DEeRNAs were identified by GSVA [27].

### Construction of the eRNA regulatory network

Pearson correlation analysis was performed for DETFs, DEeRNAs, DETGs, immune gene sets, immune cells, and hallmark pathways. Interaction pairs between DEeRNAs and other components were controlled based on a P value < 0.05 and |correlation coefficient| > 0.40. Then, with Cytoscape (v3.7.1) [28], a regulatory network was constructed based on significant interaction pairs between these components.

### ATAC-seq validation

ATAC-seq data of eRNAs were downloaded from the GEO database to determine the open chromatin region of all the DEeRNAs on the chromosome where the corresponding enhancers were located.

### Multidimensional validation

To decrease inherent deficiencies in silico, based on multivariate online databases, we chose data from tissues similar to the annulus disc, mainly tissues rich in fibroblasts, since there were no annulus disc samples available. Specifically, Pathway Card (https://pathcards.genecards.org/), KEGG, Expression Atlas [29], Genotype-Tissue Expression (GTEx) [30], String [31], and The Human Protein Atlas [32] databases were used.

### External single cell RNA-seq validation

In our study, fibroblast data derived from the Single Cell Expression Atlas (https://www.ebi.ac.uk/gxa/sc/experiments/E-HCAD-13/results) were utilized to validate the expression of DEGs and DETFs at the single-cell level.

### External ChIP-seq validation

Histone H3K27ac is involved in enhancer-specific modifications, which are critical for enhancers to promote the transcription of corresponding target genes [33]. The distribution of H3K27ac histones in T, B and K562 cells was analysed to determine enhancer-related TFs, and ChIP-seq and transcriptomic analyses were conducted to evaluate the regulatory roles of the TFs. The UCSC Genome Browser (http://genome-asia.ucsc.edu/cgi-bin/hgGateway?redirect=manual&source=genome.ucsc.edu) and the Cistrome Data Browser [19] (http://cistrome.org/) were the main instruments used to validate the chromatin accessibility of DETFs in the regions of DEeRNAs.

### hTFtarget validation

Identification of TF-target gene regulatory relationships is essential for revealing the functions of TFs and their relative regulation of the expression of target genes. Here, we used the hTFtarget database [34] (http://bioinfo.life.hust.edu.cn/hTFtarget#!/) to predict the upstream TFs of the identified DETGs.

### Hi-C validation

The Hi-C functional module of the 3D Genome Browser [35] (http://3dgenome.fsm.northwestern.edu/) was used to identify the topologically associating domains (TADs) of identified DEeRNAs through analysis of Hi-C data [36], which enabled us to validate the potential regulatory events between identified DEeRNAs and DETGs.

### Statistical analysis

In our study, all statistical analysis processes were performed by R software version 3.6.1 (Institute for Statistics and Mathematics, Vienna, Austria). Statistical results were expressed as mean ± standard deviation (M ± SD). Data comparison of the two groups were analyzed using the Wilcoxon rank-sum test. Two-tailed p value < 0.05 was considered statistically significant. GraphPad Prism 7.0 was used to plot line charts.

## Results

### Identification of DETGs and DEeRNAs

**Fig. 1:**
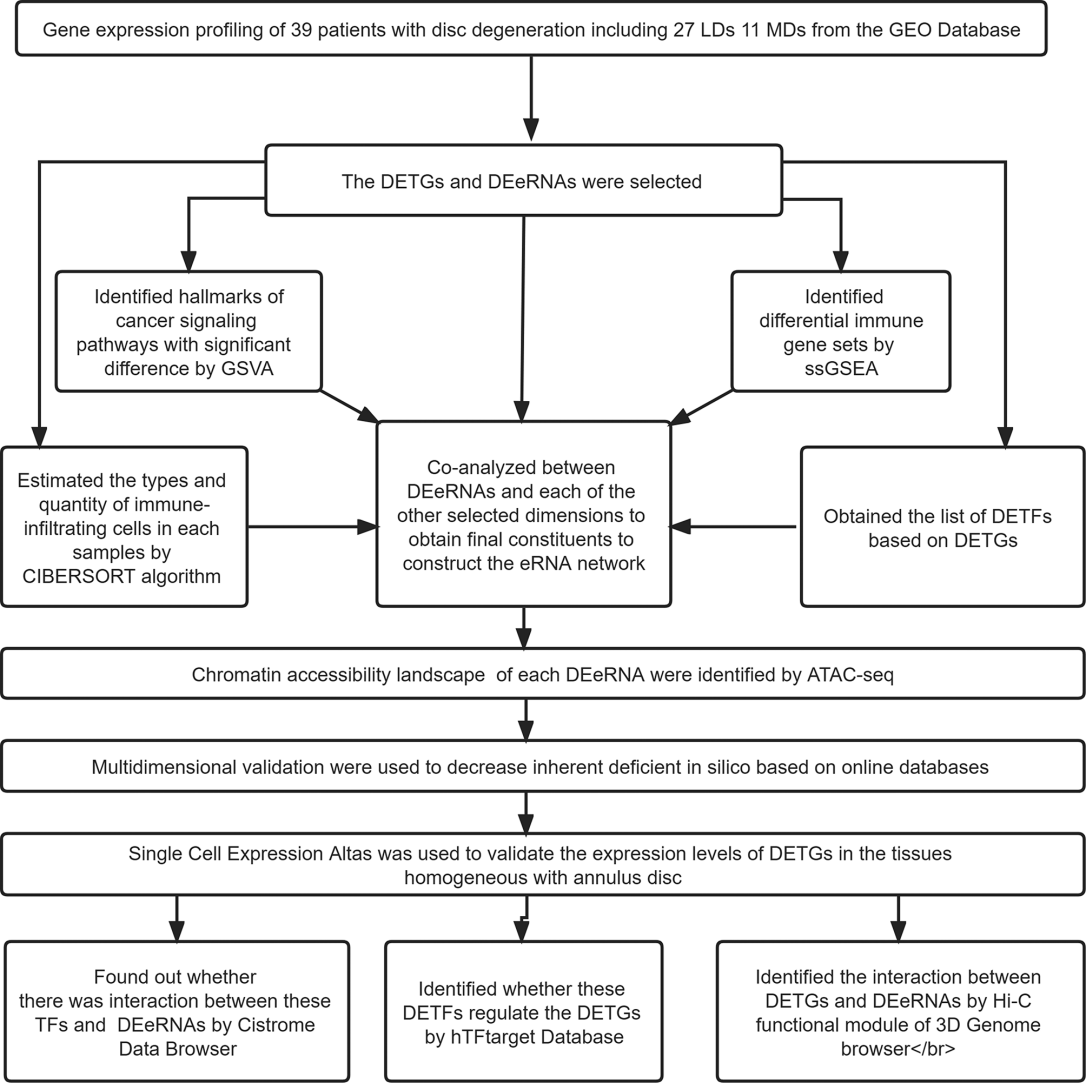
Flow diagram of the analytical process

**Fig. 2:**
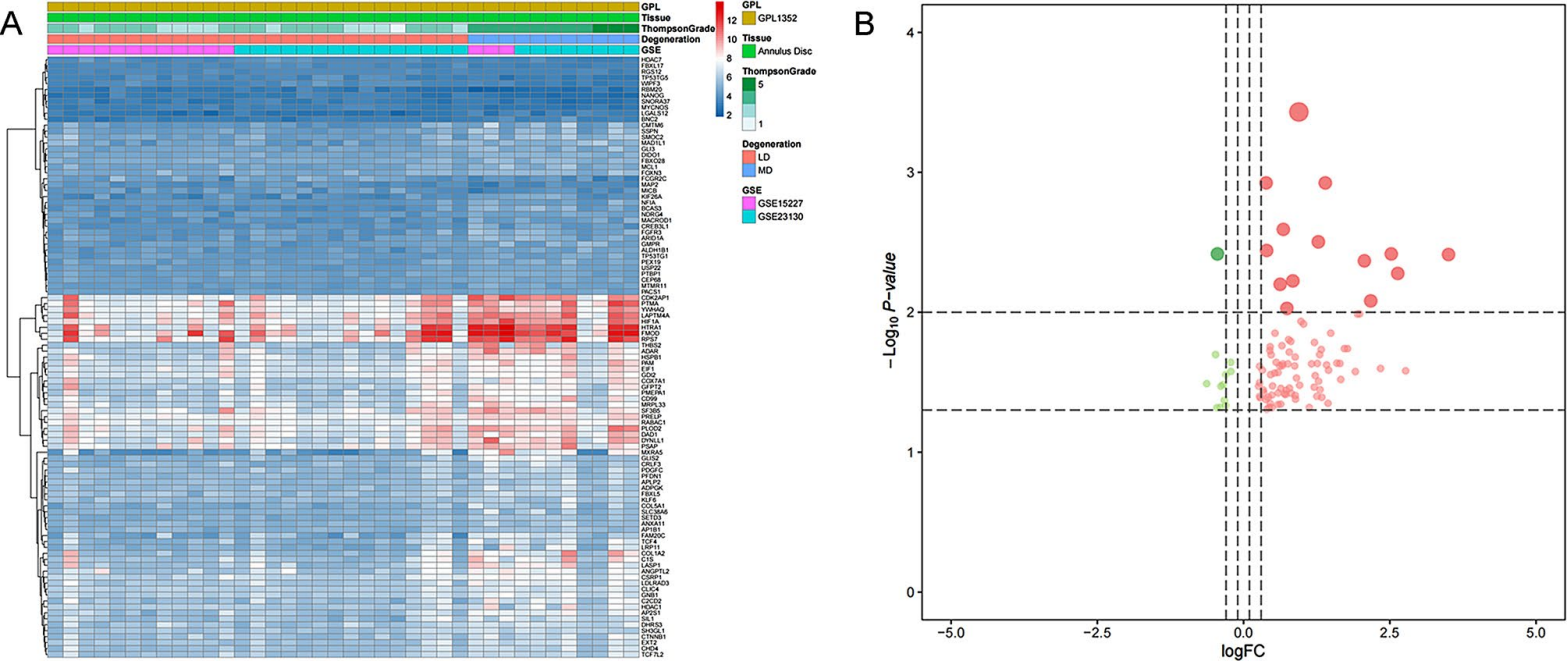
Heatmap (**A**) and volcano plot (**B**) of eRNAs from the samples. Red and blue indicate the LD samples and MD samples, respectively. The green and red dots represent significantly differential expression

**Fig. 3:**
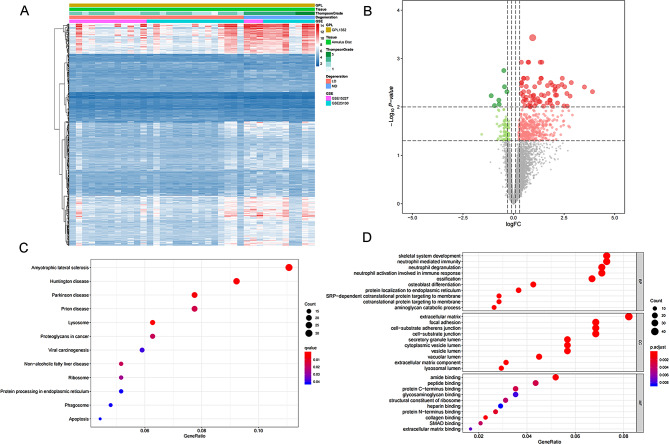
(**A**) Differentially expressed genes between LDs and MDs of annulus disc samples; red and blue indicate LD samples and MD samples, respectively. Volcano plot for genes from the samples (**B**); green and red dots represent significantly differential expression. Bubble plot for KEGG (**C**) and GO (**D**) enrichment analysis. Abbreviations: Gene Oncology (GO), Kyoto Encyclopedia of Genes and Genomes (KEGG)

The complete process of this research is illustrated in the flow chart (Fig. 1). The clinical information of the 38 IVDD patients is shown in Table S1. A total of 101 DEeRNAs (89 upregulated, 12 downregulated) and 555 DEGs (457 upregulated, 98 downregulated) were identified between LDs and MDs with a threshold of |log2 FC| > 1 and FDR P value < 0.05, as shown in heatmaps and volcano plots of DEeRNAs (Fig. 2A and B) and DEGs (Fig. 3A and B). The list of DEGs targeted by these DEeRNAs was acquired from the supplementary material of Zhao Zhang et al. [17]. Based on these results, a total of 75 DETGs were identified, and their expression was demonstrated via a heatmap (Figure S1 A) and volcano plot (Figure S1 B).

### Functional enrichment analysis

Based on GO and KEGG analyses of the potential functional mechanism of DETGs, the most important GO terms in the BP, CC, and MF of GO analysis were skeletal system development, extracellular matrix, and amide binding, respectively (Fig. 3C). The most significant KEGG pathway was amyotrophic lateral sclerosis (Fig. 3D).

### Correlation analysis of DEeRNAs and DETGs

A total of 141 regulatory relationships between DEeRNAs and DETGs were identified by correlation analysis with the abovementioned thresholds of a |correlation coefficient|> 0.300 and a *p* value < 0.001. Based on the significant regulatory relationships of DEeRNAs and DETGs, complement C1s (C1S) (eRNA, enhancer ID: 12:6989331–6,995,331) and chromodomain helicase DNA binding protein 4 (CHD4) (DETG) were extracted for subsequent analysis (*R* = 0.915, *P* < 0.001, positive).

### Identification of DETFs and correlation analysis of DEeRNAs and DETFs

**Fig. 4:**
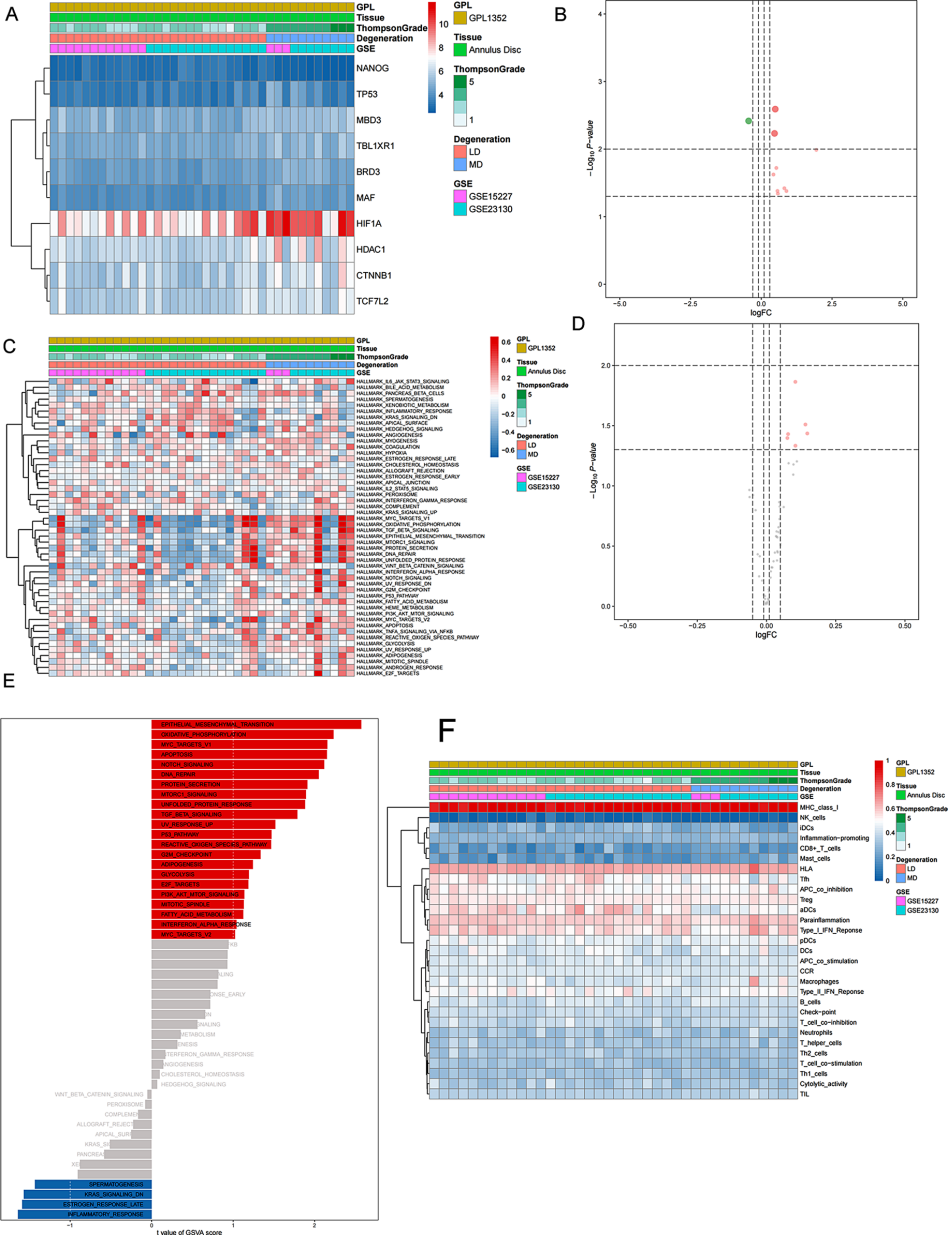
(**A**) DETFs between LD samples and MD samples; red and blue indicate LD samples and MD samples, respectively. Volcano plot (**B**) for DETFs from samples; green and red dots represent significantly differential expression. Heatmap (**C**) and volcano plot (**D**) showing the 50 hallmark gene sets acquired from MsigDB that were identified by GSVA between LD samples and MD samples. Heatmap (**E**) showing the GSVA scores of hallmark signalling pathways. Heatmap (**F**) of significant immune cells and signalling pathways related to immunological characteristics according to ssGSEA. Abbreviations: Quantitative gene set variation analysis (GSVA)

Ten DETFs were identified by the limma package with the thresholds of |log2 FC| > 1 and FDR < 0.05, as shown in the heatmap (Fig. 4A) and volcano plot (Fig. 4B). A total of 63 regulatory relationships between DEeRNAs and DETFs were identified by correlation analysis based on the thresholds of a |correlation coefficient|> 0.300 and a P value < 0.001. The most significant DETF related to C1S was transcription factor 7 like 2 (TCF7L2) (*R* = 0.876, *P* < 0.001, positive), followed by CTNNB1 (*R* = 0.831, *P* < 0.001, positive) and TBL1XR1 (*R* = 0.825, *P* < 0.001, positive).

### GSVA, ssGSEA, correlation analysis of DEeRNAs and hallmarks of cancer signalling pathways

Based on the gene expression of 27 LDs and 11 MDs, the enrichment levels of 50 hallmark pathways were calculated using GSVA, and the results are shown in a heatmap (Fig. 4C) and volcano plot (Fig. 4D). Specifically, based on the correlation analysis, EPITHELIAL_MESENCHYMAL_TRANSITION (*R* = 0.888, *P* < 0.001, positive), OXIDATIVE_PHOSPHORYLAION (*R* = 0.875, *P* < 0.001, positive) and MYC_TARGETS_V1 (*R* = 0.845, *P* < 0.001, positive) were significantly related to C1S in IVDD samples. The specific t value of the GSVA score of each signalling pathway between IVDD samples and nondegenerated samples is illustrated in Fig. 4E. In addition, 26 hallmark pathways were extracted for further analysis. The enrichment levels of 29 immune-associated gene sets were evaluated by ssGSEA, and the results are shown in Fig. 4F. Specifically, MHC class I, NK cells and iDCs were significantly enriched in IVDD samples.

### Immune infiltration analysis and correlation analysis of DEeRNAs and immune cells

**Fig. 5:**
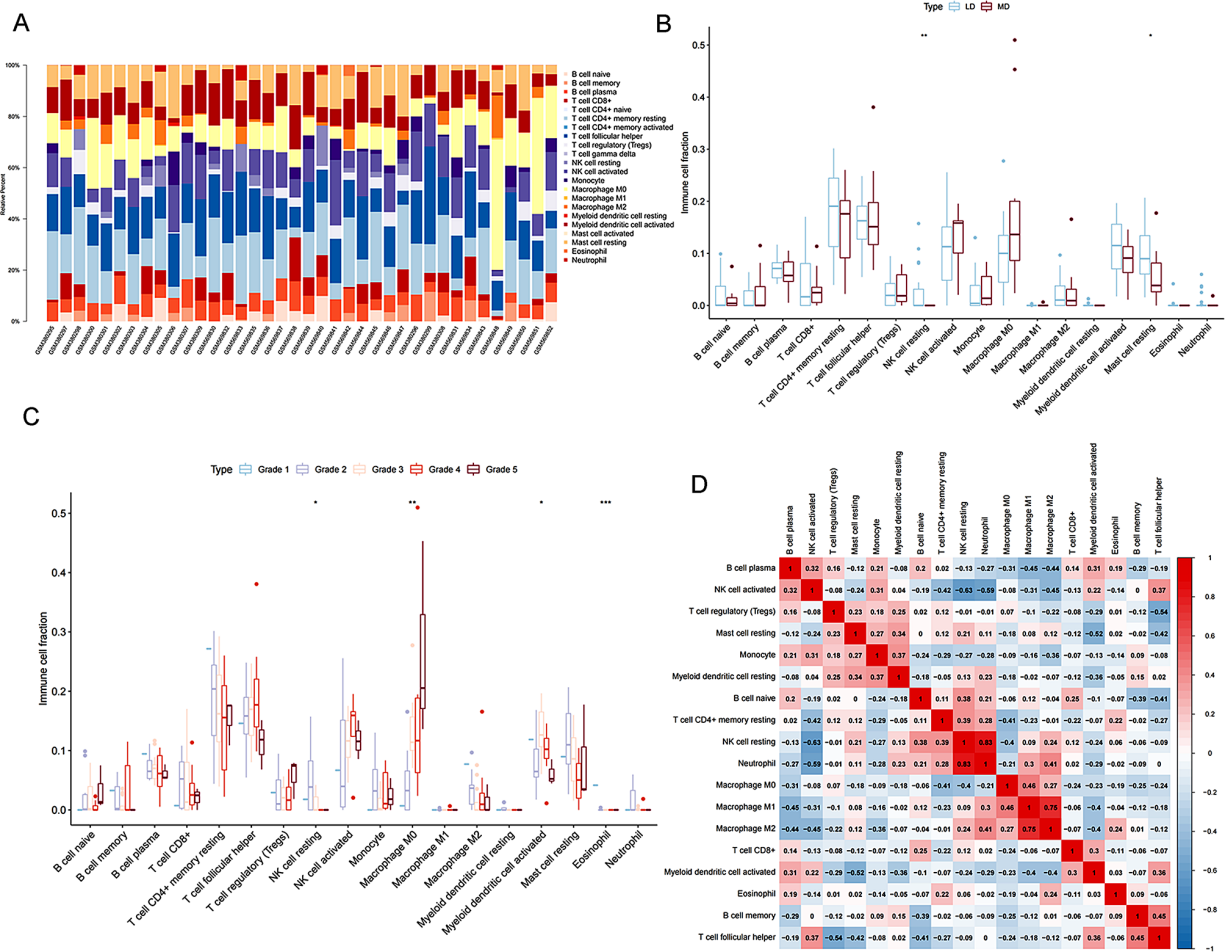
(**A**) The type and composition of immune cells estimated by the CIBERSORT algorithm in the samples. (**B**) The type of immune cells with significant differences between LD samples and MD samples; red and blue indicate MD samples and LD samples, respectively. (**C**) The types of immune cells with significant differences between the grades. (**D**) The coexpression patterns among identified immune cells

The infiltration levels of 22 types of immune cells in each sample were identified through the CIBERSORT algorithm based on relative subsets of RNA transcripts. The levels of immune cells in each sample are shown in the heatmap (Fig. 5A). A significant correlation between immune cells and IVDD was revealed by nonparametric tests (Fig. 5B and C), which indicated that the quantity of resting NK cells and resting mast cells in LDs was greater than that in MDs, and there were significant differences in the quantity of resting NK cells, M0 macrophages, activated myeloid dendritic cells and eosinophils to varying degrees in IVDD. The coexpression patterns among the identified immune cells are shown in Fig. 5D.

Based on the coanalysis of C1S and 22 types of immune cells/pathways, we found that M0 macrophages (*R* = 0.520, *P* < 0.01, positive), resting mast cells (*R*=-0.441, *P* < 0.05, negative), and resting CD4^+^ memory T cells (*R*=-0.375, *P* < 0.05, negative) were involved in IVDD progression.

### Construction of the regulatory network

**Fig. 6:**
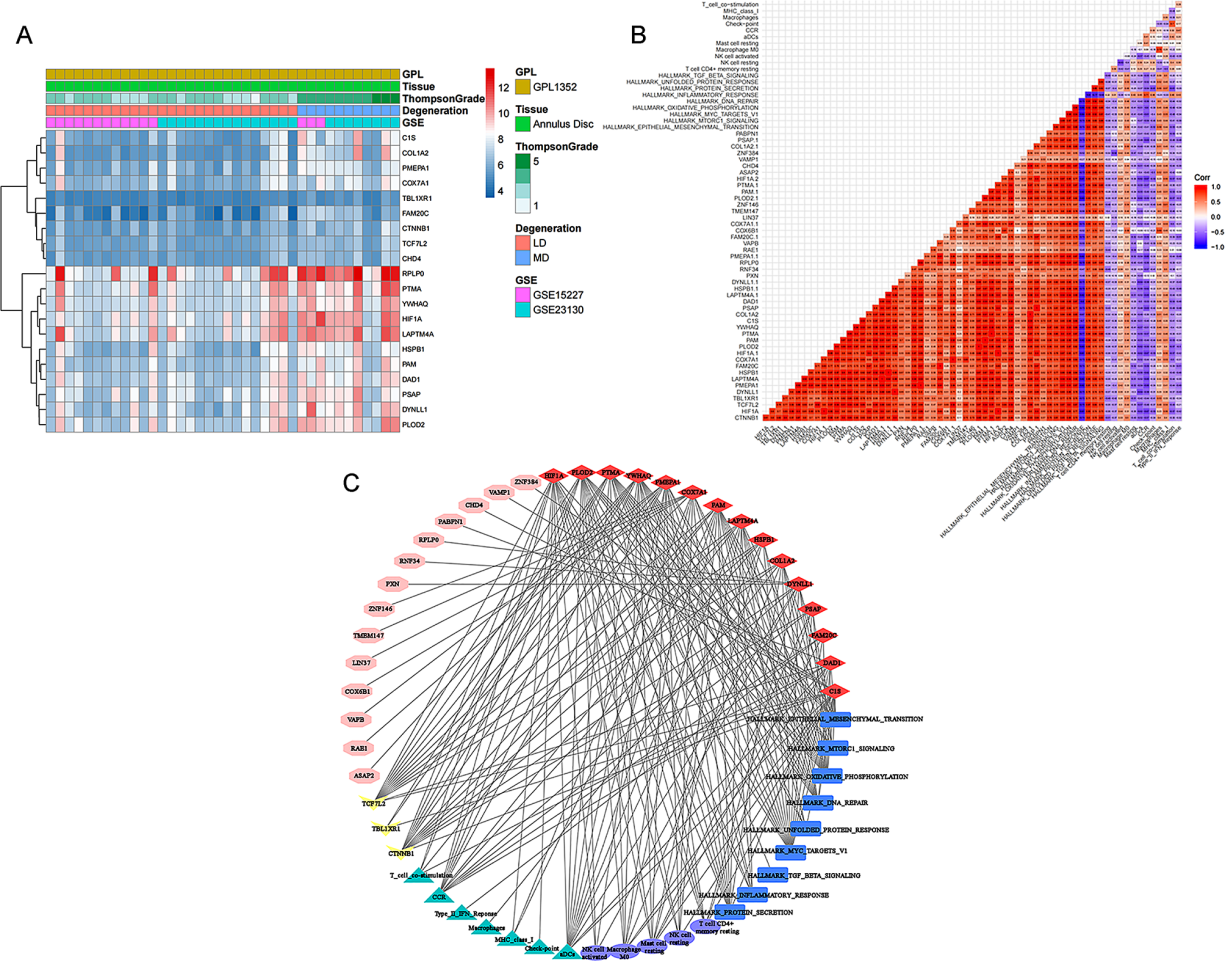
Construction of the DEeRNA network. Heatmap (**A**) for DEGs, DETFs and DEeRNAs on the final list from the selection process. Coheatmap (**B**) of all 6 dimensions and network (**C**) of all 6 dimensions on the final list, including eRNAs (red diamonds), hallmarks of cancer signalling pathways according to GSVA (blue rectangles), immune gene sets according to ssGSEA (green triangles), immune cells according to CIBERSORT (purple ellipses), DEeRNA target genes (pink hexagons) and DETFs (yellow arrowheads)

A total of 15 DEeRNAs, 14 DETGs, 3 DETFs, 5 immune cells, 9 hallmarks of cancer signalling pathways, and 7 immune-associated gene sets showing high correlations were extracted for subsequent analysis. The expression levels of these transcriptome components are illustrated in the heatmap (Fig. 6A). The components in the regulatory network are listed in Table S2. We performed correlation analysis between these eRNAs and the remaining 6 components, and a regulatory network was constructed based on significant correlation pairs (Fig. 6B). The results of the coexpression analysis of all 53 components in the network are displayed in Fig. 6C.

### ATAC-seq validation

**Fig. 7:**
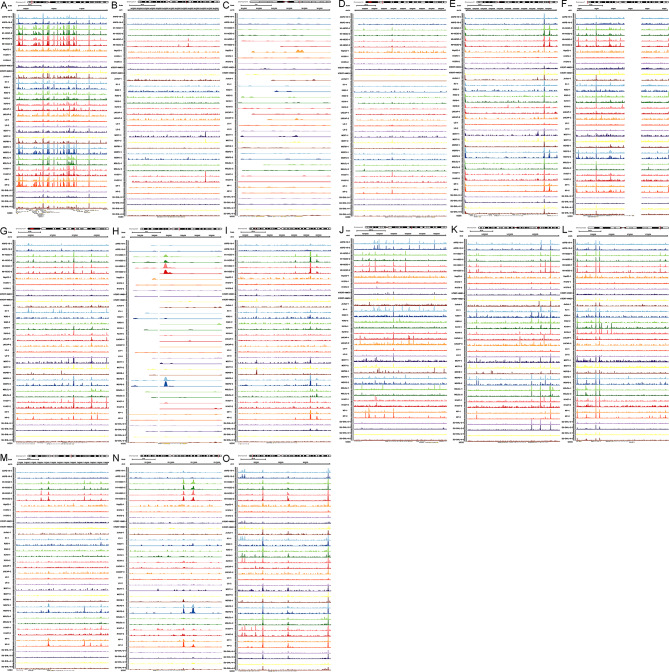
IGV plots of the chromatin accessibility landscape of all 15 DEeRNAs in the samples: C1S (**A**), COL1A2 (**B**), COX7A1 (**C**), DAD1 (**D**), DYNLL1 (**E**), FAM20 (**F**), HIF1A (**G**), HSPB1 (**H**), LAPTM4A (**I**), PAM (**J**), PLOD2 (**K**), PMEPA1 (**L**), PSAP (**M**), PTMA (**N**), and YWHAQ (**O**). The presence of colourful peaks indicates chromatin accessibility

Figure 7 shows the open chromatin region of all 15 DEeRNAs on the chromosome where the corresponding enhancer is located, and the presence of colourful peaks indicates chromatin accessibility.

### Multidimensional validation

To decrease the bias of different algorithms and databases, we used multivariate external databases. Based on Pathway Card and KEGG analyses, PXN was related to HALLMARK_TGF_BETA_SIGNALING and HALLMARK_MTORC1_SIGNALING, and COX6B1 was involved in the oxidative phosphorylation signalling pathway, which is related to DETFs, CTNNB1 and TCF7L2. CTNNB1 also plays a role in the TGF-β signalling pathway. Moreover, DETFs, CTNNB1, TCF7L2 and TBL1XR1 are vitally important for the Wnt signalling pathway. Genotype-Tissue Expression (GTEx) was used to identify the expression levels of the DEGs and DETFs in multiple normal tissues (Figure S2). DepMap (Figure S3) shows the expression levels of DETGs and DETFs in multiple cancers, including samples homogeneous to the annulus disc, such as chondrosarcoma, and fibroblasts in multiple tissues.

### External single cell RNA-seq validation

**Fig. 8:**
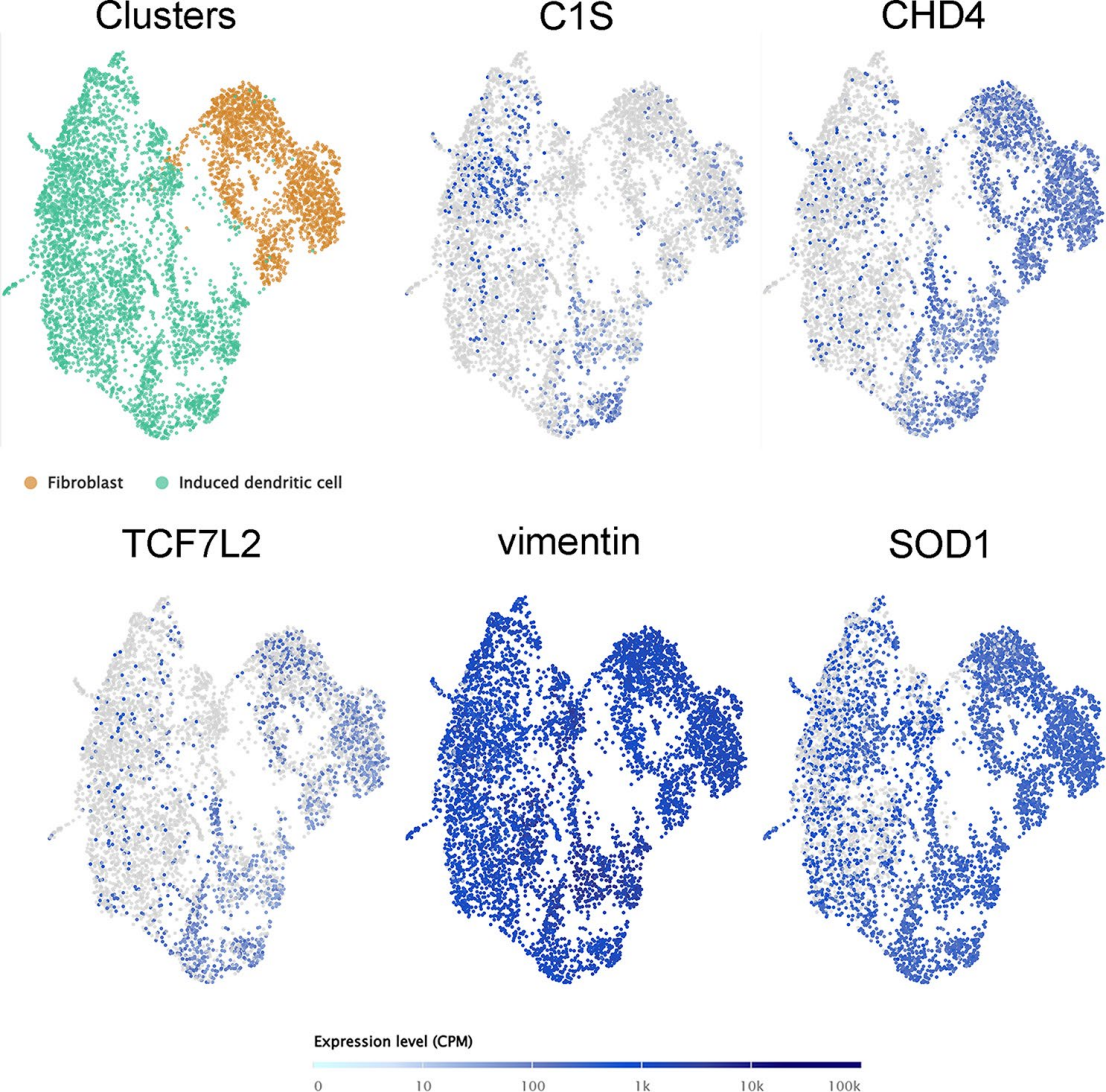
External single-cell RNA-seq validation by the Single Cell Expression Altas. A total of 13 key DEGs and 3 DETFs were expressed in the lung fibrosis samples, especially COX6B1, RPLP0, TMEM147, CHD4 and PXN, which were highly expressed

Based on the abundance of several subclusters of fibroblasts expressing high levels of fibroblast signature genes, such as CEMIP, AKR1C1, MGP, COMP, DNER, and MELTF [37, 38], the data of fibroblasts derived from the Single Cell Expression Atlas were used to validate the expression of DEGs and DETFs at the single-cell level. Importantly, in addition to CTNNB1, C1S and CHD4, the key gene vimentin in EPITHELIAL_MESENCHYMAL_TRANSITION and the key gene superoxide dismutase 1 (SOD1) in OXIDATIVE_PHOSPHORYLAION were coexpressed in fibroblasts (Fig. 8).

### External ChIP-seq validation

**Fig. 9:**
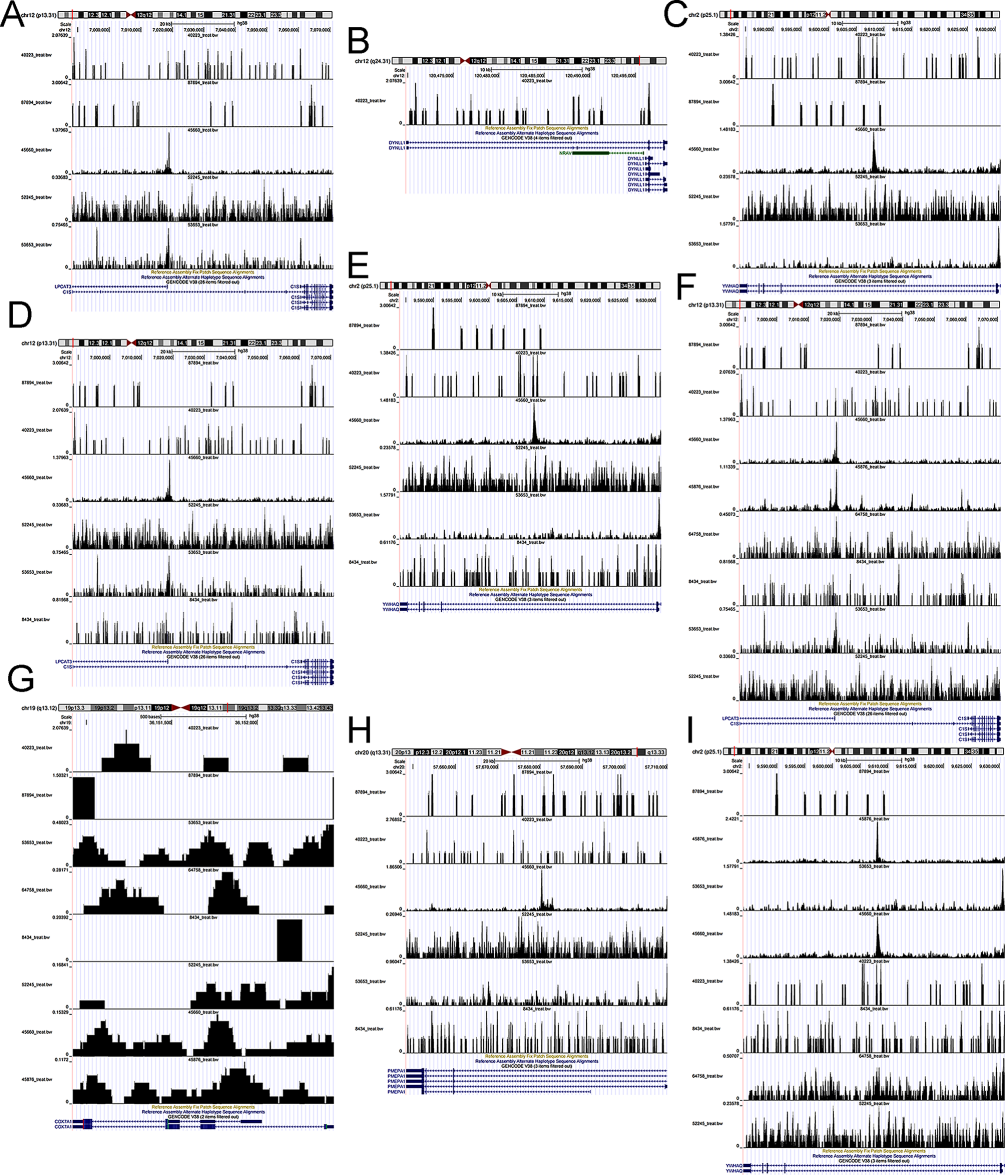
Chromatin accessibility of DETFs. The presence of black peaks indicates chromatin accessibility. (**A, B, C**) CTNNB1 binds to C1S, DYNLL1 and YWHAQ. (**D, E**) Chromatin accessibility of TBL1XR1 at the chromosomal locations of C1S and YWHAQ. (**F, G, H, I**) Chromatin accessibility of TCF7L2 at the chromosomal locations of C1S, COX7A1, PMEPA1 and YWHAQ

The UCSC Genome Browser was used in our study to determine the locations of the DEeRNAs, and the chromatin accessibility (Fig. 9) of CTNNB1 (T lymphocytes), TCF7L2 (K562) and TBL1XR1 (B lymphocytes) was subsequently determined via the Cistrome Data Browser.

### TF target validation

**Fig. 10:**
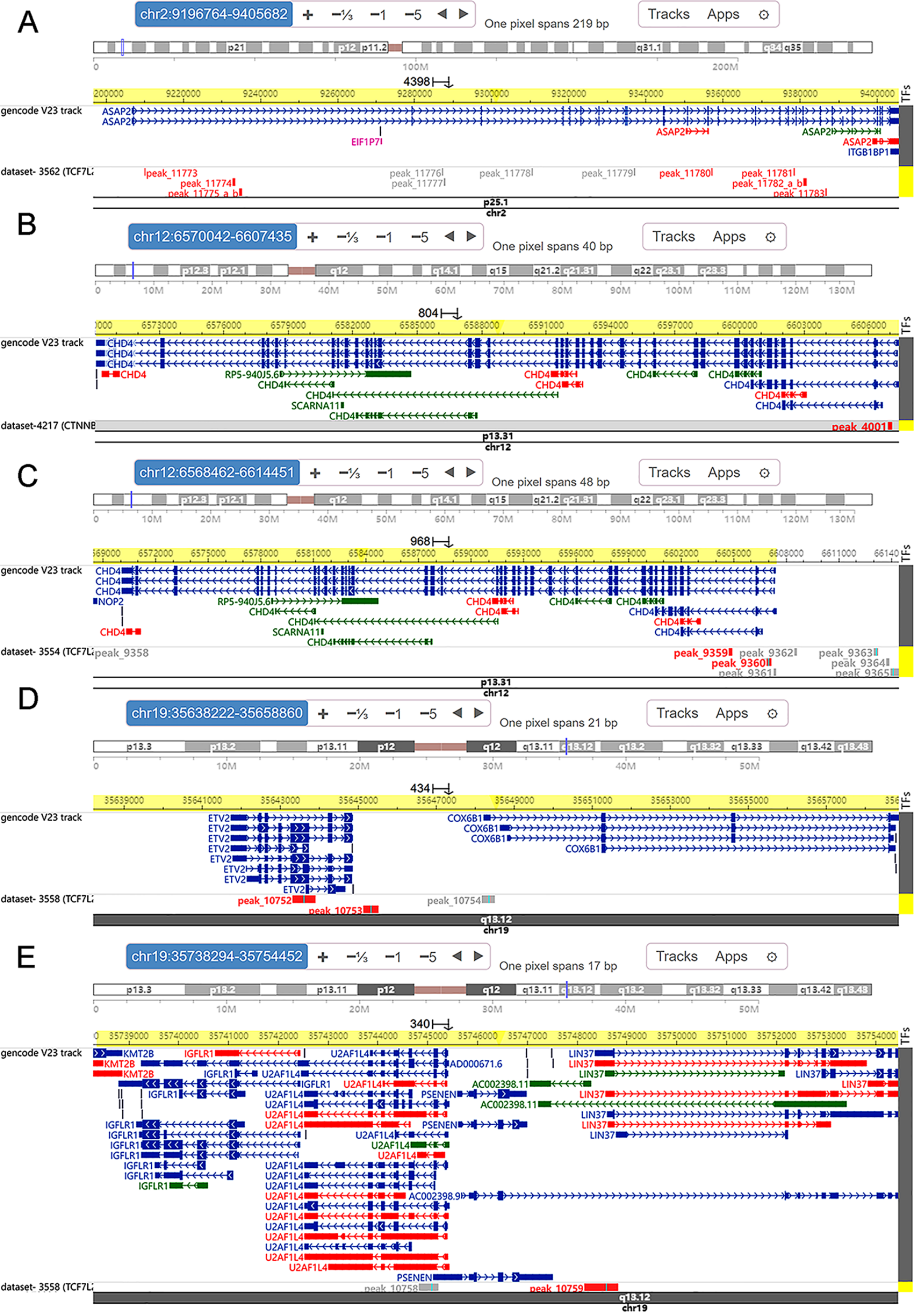
Validation of external TF targets via the hTFtarget database. Regulation and interaction locations of TCF7L2-ASAP2 (**A**), CTNNB1-CHD4 (**B**), TCF7L2-CHD4 (**C**), TCF7L2-COX6B1 (**D**) and TCF7L2-LIN37. The blue, green and red fishbone lines represent the coding, noncoding and problem states of genes, respectively. The short lines at the bottom of each graph indicate the position at which DETFs bind to genes, and the redness depth of the DETFs indicates the binding intensity

Using the hTFtarget database for the 3 DETFs and 15 DEGs in the network, we identified that CTNNB1 targets CHD4, and TCF7L2 targets CHD4, COX6B1, LIN37 and ASAP2 (Fig. 10). Moreover, COX6B1 was found to be the common target DEG for TBL1XR1 and TCF7L2. In the hTFtarget database, based on the GSM935574 and 935,678 data, there was a strong association between TBL1XR1 and TCF7L2 in the K562 cell line derived from bone marrow [the relative importance (RI) score = 37.68] (MAX value: 100, a higher score means that the two TFs have a stronger coassociation).

### Hi-C validation

**Fig. 11:**
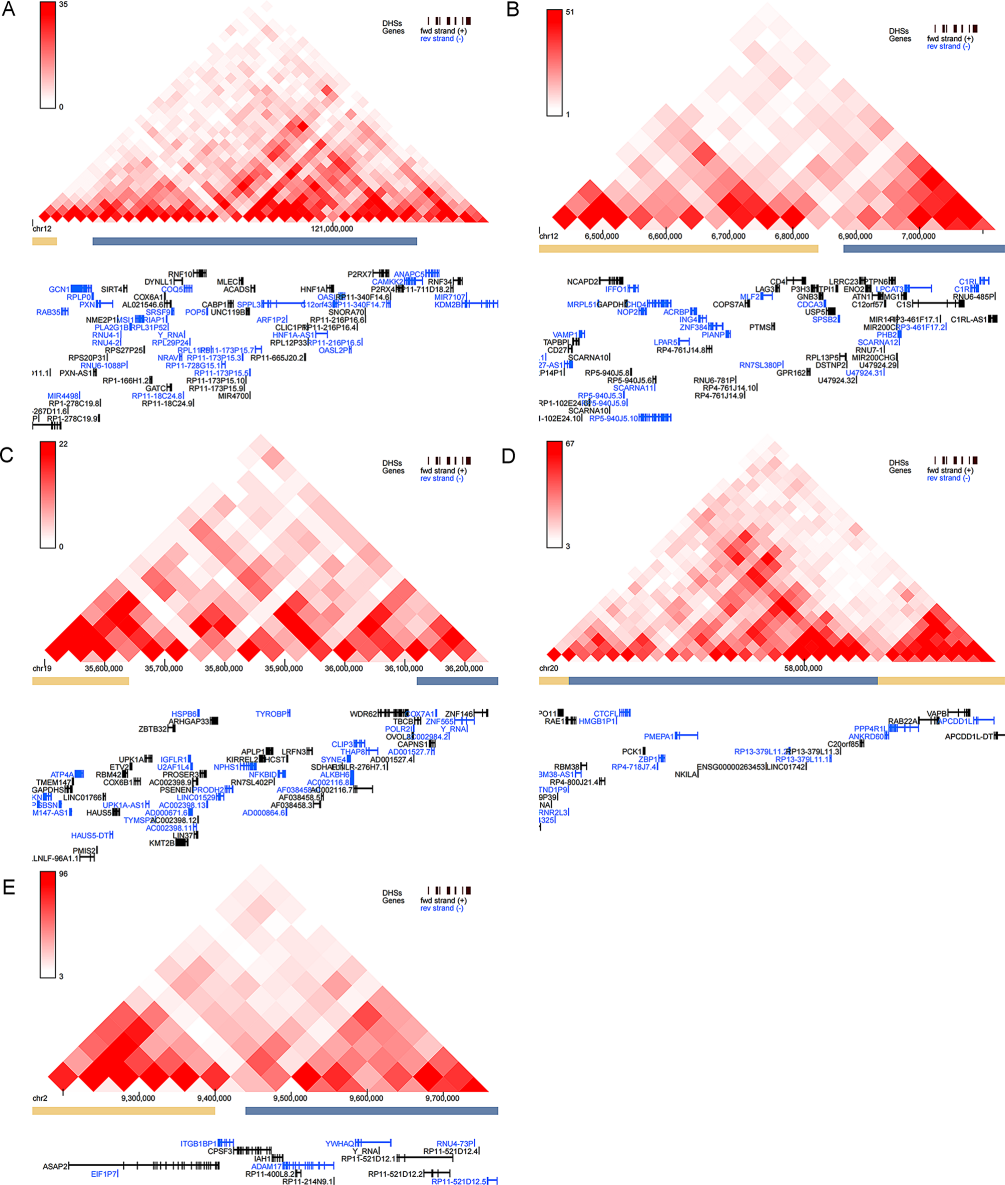
Analysis of Hi-C data from IMR90_Flavopiridol using the 3D Genome Browser. DYNLL1 and PXN, RNF34 and RPLP0 (**A**); C1S and CHD4, VAMP1, ZNF384 (**B**); COX7A1 and COX6B1, LIN37, TMEM147 and ZNF146 (**C**); PMEPA1 and RAE1, VAPB (**D**); YWHAQ and ASAP2 can interact with each other. The colour of the point where the upper corner of the triangle is located reflects the interaction of the genes through which the vertical line connects the two lower corners of the grey triangle

IMR90 is a type of fibroblast derived from foetal lung tissue that is histologically homogeneous to the annulus disc. Moreover, flavopiridol is a broad-spectrum inhibitor of cyclin-dependent kinases (CDKs) [39]. This finding is consistent with the significant upregulation of CDK 2-associated protein 1 (CDK2AP1) (logFC = 2.525, adjusted P value = 0.004) between the LD and MD groups in our study. CDK2AP1 is associated with CDK2 and is thought to suppress CDK2 function by sequestering monomeric CDK2 and then targeting CDK2 for proteolysis. Therefore, we used Hi-C sequencing of the IMR90_Flavopiridol cell line to validate the interaction between DETGs and DEeRNAs. We found that C1S and CHD4, VAMP1, ZNF384; DYNLLI and PXN, RNF34, RPLP0; PMEPA1 and RAE1, VAPB; COX7A1 and COX6B1, LIN37, TMEM147, ZNF146; YWHAQ and ASAP2 were close to and interacted with each other in three-dimensions (Fig. 11).

## Discussion

We detected correlations between C1S and CTNNB1, C1S and CHD4, C1S and M0 macrophages, and C1S and OXIDATIVE_PHOSPHORYLAION. A total of 53 components, 14 DEGs, 15 eRNAs, 3 DETFs, 5 immune cells, 9 hallmarks of cancer signalling pathways, and 7 immune gene sets, were selected to construct the regulatory network. In addition to the C1S-CTNNB1-CHD4 axis, 20 other interactive DEeRNA-DETF-DEG axes are involved in IVDD exacerbation (Table 1), among which CTNNB1 targets CHD4 and TCF7L2 targets CHD4, COX6B1, LIN37 and ASAP2, as proven by the hTFtarget database. With respect to cis-acting elements, recent studies have shown that after being transcribed by enhancer units, eRNAs activate their target genes via overtranscription. Specifically, eRNAs located around the TFBS of target genes subsequently recruit RNA polymerase II, transcription factors or coactivators/transcriptional complexes to bind target gene TFBSs, which ultimately leads to the upregulation of their target genes. In our study, based on the functional pattern of eRNAs and the 3D interaction among these DEeRNAs, DETFs and DETGs, we speculated that C1S (eRNA location: 12:6989331–6,995,331) recruits CTNNB1 to increase the expression of CHD4 in IVDD.

**Table 1:** 20 interactive “DEeRNA-DETF-DEG” axises

eRNA	TF	DEG
DYNLLI	CTNNB1	PXN
		RNF34
		RPLP0
PMEPA1	TCF7L2	RAE1
		VAPB
C1S	CTNNB1	CHD4
	TBL1XR1	VAMP1
	TCF7L2	ZNF384
COX7A1	TCF7L2	COX6B1
		LIN37
		TMEM147
		ZNF146
YWHAQ	CTNNB1	ASAP2
	TBL1XR1	
	TCF7L2	

The intervertebral disc, especially the nucleus pulposus, is an immune-privileged tissue. An autoantigen triggers the immunological cascade once the physiological barrier is disrupted [40]. The impact of the complement system on IVDD was demonstrated in recent work. The complement system plays a role in IVDD by forming the terminal complement complex (TCC), an activator of inflammatory processes and cell lysis that has been shown to be highly deposited in degenerated intervertebral discs [41, 42]. In our study, we found that the gene fragment overlapping the DNA location of C1S, worsening IVDD, occurred not only by forming a TCC but also by acting as an eRNA, regulating some IVDD-related genes.

β-Catenin, encoded by CTNNB1, is an adhesion junction component that, together with cadherin and α-catenin, forms the adherens junction complex and plays an important role in the construction and maintenance of epithelial cell layers by regulating cell growth and intercellular adhesion [43]. Macrophages participate in the degradation and remodelling of the ECM through the production of MMPs (MMP-7, 9, 12) and A disintegrin and metalloproteinase with thrombospondin motifs [44–47]. Furthermore, the chondrocytes and chondrocyte-like cells in the IVD secrete MMP-1 [48], MMP-2 [49], MMP-3 [50], MMP-9 [51] and MMP-13 [52]. The decomposition of the ECM is an important change in the development of IVDD. Interestingly, MMP-1, MMP-2, MMP-3, MMP-7, MMP-8, MMP-9, MMP-10, MMP-11, MMP-13, MMP-14, MMP-16, MMP-17, MMP-15, MMP-24, MMP-25, and MMP-27 were related to CTNNB1 (Figure S4), and these genes were verified to be involved in the pathogenesis of IVDD except for the latter four. These findings suggest that the eRNA C1S may recruit CTNNB1 to play a role in the mechanism of IVDD involving macrophages and MMPs.

CHD4 is an ATP-dependent chromatin remodeller involved in the epigenetic regulation of gene transcription, DNA repair, and cell cycle progression [53]. Recent studies have revealed a new function of CHD4 related to cell bioenergetics. CHD4 suppressed the expression of protein arginine deiminase 1 (PADI1) and PADI3, which are able to regulate citrullination of arginine residues of the allosterically regulated glycolytic enzyme pyruvate kinase M2 (PKM2) [54]. As a rate-limiting enzyme in glycolysis, citrullinated PKM2 slows glycolysis and alters the cell-bioenergetic balance between oxidative phosphorylation and glycolysis, which triggers and promotes the progression of fibrosis. Based on our findings on the roles of eRNAs and the C1S-CTNNB1-CHD4 axis in IVDD, we speculated that the eRNA C1S recruited CTNNB1 and upregulated the expression of CHD4 in IVDD and then suppressed glycolysis by modulating PKM2 and activating the oxidative phosphorylation process, thus resulting in insoluble collagen fibre deposits and leading to the genesis and exacerbation of IVDD. Deficience of experimental validation is one of the limitation of our study, and the subsequent validation of interactions among DEeRNAs, DETFs and DEGs, especially the C1S-CTNNB1-CHD4 axis in space in IVDD is worthy to carry out.

## Conclusion

Based upon the results of our study, we theorize that the C1S-CTNNB1-CHD4 axis plays a vital role in IVDD exacerbation. Specifically, C1S recruits CTNNB1 and upregulates the expression of CHD4 in IVDD, and subsequently, CHD4 suppresses glycolysis and activates oxidative phosphorylation, thus generating insoluble collagen fibre deposits and leading to the progression of IVDD. Overall, these DEeRNAs could comprise promising therapeutic targets for IVDD due to their high tissue specificity.

### Electronic supplementary material

Below is the link to the electronic supplementary material.

Supplementary Material 1

Supplementary Material 2

Supplementary Material 3

Supplementary Material 4

Supplementary Material 5

Supplementary Material 6

## Data Availability

Publicly available datasets were analyzed in this study. This data can be found here: Gene Expression Omnibus (GSE15227, GSE23130, GSM1195331, GSM935653, GSM722419, GSE139099) (GEO, https://www.ncbi.nlm.nih.gov/geo/), ImmPort database (http://www.immport.org/), and Cistrome Cancer database (http://cistrome.org/).
