# Supplementary figures and images for: Identification of key eRNAs for intervertebral disc degeneration by integrated multinomial bioinformatics analysis
Source: BMC Musculoskelet Disord. 2024 May 4;25:356. doi: 10.1186/s12891-024-07438-6 (PMC11069191; doi:10.1186/s12891-024-07438-6)

**
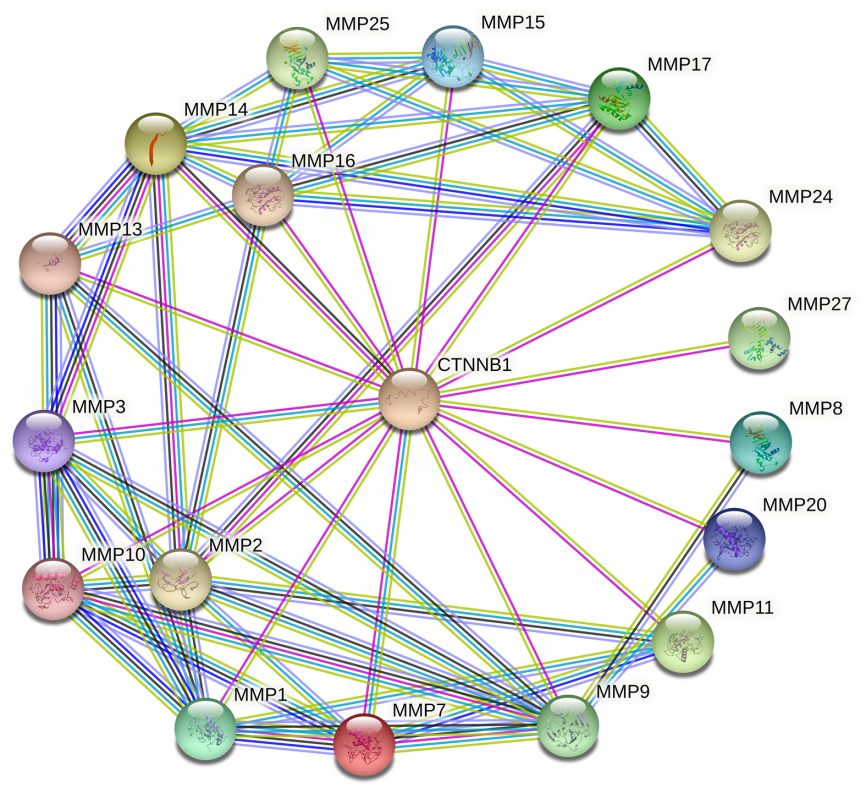
**

**Figure S4** The relationship between CTNNB1 and MMPs.

Supplement: Supplementary file 1 — Supplementary Material 1 [file 12891_2024_7438_MOESM1_ESM.docx]

**
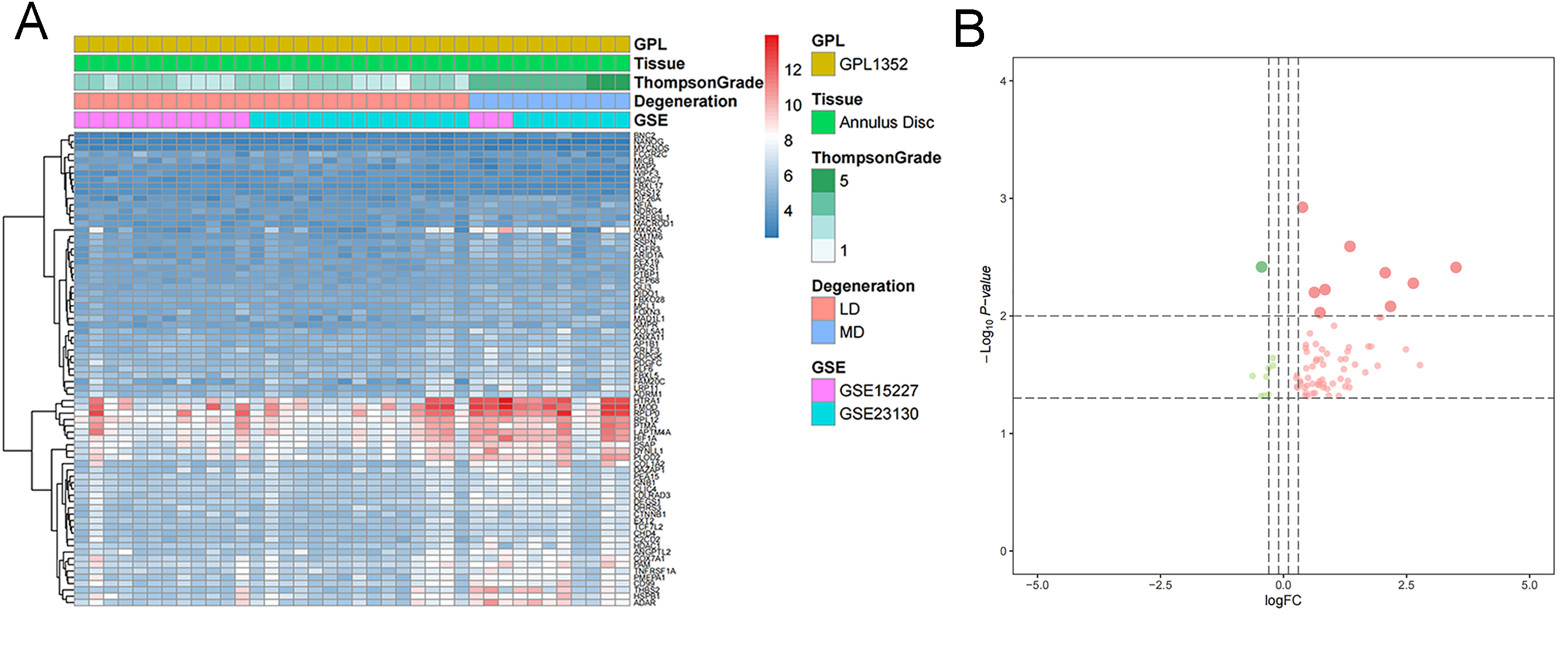
**

**Figure S1** Heatmap (A) and volcano plot (B) for the DEGs related to selected eRNAs.

Supplement: Supplementary file 2 — Supplementary Material 2 [file 12891_2024_7438_MOESM2_ESM.docx]

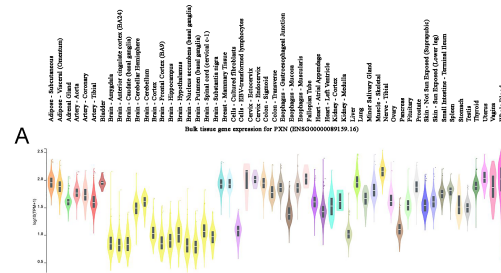

Supplement: Supplementary file 5 — Supplementary Material 5 [file 12891_2024_7438_MOESM5_ESM.pdf]
