# Supplementary material for: Identification of key eRNAs for intervertebral disc degeneration by integrated multinomial bioinformatics analysis
Source: BMC Musculoskelet Disord. 2024 May 4;25:356. doi: 10.1186/s12891-024-07438-6 (PMC11069191; doi:10.1186/s12891-024-07438-6)
Supplement: Supplementary file 3 — Supplementary Material 3 [file 12891_2024_7438_MOESM3_ESM.docx]

**
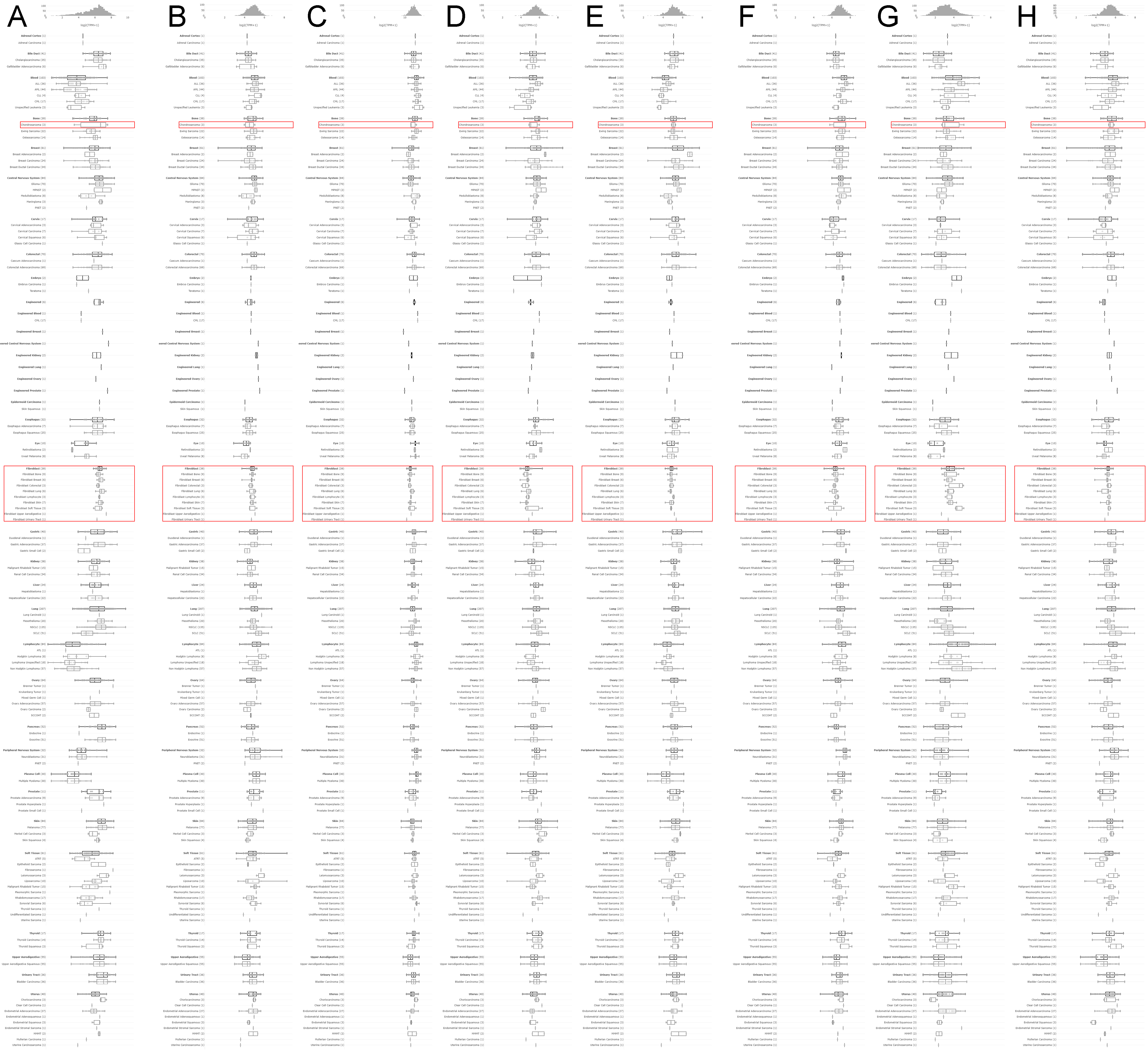
**

**Figure S3** The expression levels and perturbation effects of DEeRNAs in multiple kind of cells including chondrocytes and fibroblasts.
