# Supplementary material for: Identification of key eRNAs for intervertebral disc degeneration by integrated multinomial bioinformatics analysis
Source: BMC Musculoskelet Disord. 2024 May 4;25:356. doi: 10.1186/s12891-024-07438-6 (PMC11069191; doi:10.1186/s12891-024-07438-6)
Supplement: Supplementary file 4 — Supplementary Material 4 [file 12891_2024_7438_MOESM4_ESM.docx]

| **Variables** | **Total Specimen(N=38)** |
| --- | --- |
| **Spinal segment** |  |
| C5-C7 | 2(5.3%) |
| L2-S1 | 12(31.6%) |
| Lumar | 1(2.6%) |
| Unknown | 23(60.5%) |
| **Tissue** |  |
| Annulus Disc | 38(100%) |
| **Tissue source** |  |
| CHTN specimen | 16(42.1%) |
| Surgical specimen | 22(57.9%) |
| **Degree of degeneration** |  |
| Lightly degenerated disc | 27(71.1%) |
| Moderately degenerated disc | 11(28.9%) |
| **Thompson Grade** |  |
| Grade 1 | 1(2.6%) |
| Grade 2 | 10(26.3%) |
| Grade 3 | 16(42.1%) |
| Grade 4 | 8(21.1%) |
| Grade 5 | 3(7.9%) |

**Table S1** Details on disc tissues.
