# Supplementary material for: Identification of key eRNAs for intervertebral disc degeneration by integrated multinomial bioinformatics analysis
Source: BMC Musculoskelet Disord. 2024 May 4;25:356. doi: 10.1186/s12891-024-07438-6 (PMC11069191; doi:10.1186/s12891-024-07438-6)
Supplement: Supplementary file 6 — Supplementary Material 6 [file 12891_2024_7438_MOESM6_ESM.docx]

Table S2. The components in the regulatory network.

| Component | Type |
| --- | --- |
| CTNNB1 | TF |
| TCF7L2 | TF |
| TBL1XR1 | TF |
| DYNLL1 | eRNA |
| PMEPA1 | eRNA |
| LAPTM4A | eRNA |
| HSPB1 | eRNA |
| FAM20C | eRNA |
| COX7A1 | eRNA |
| HIF1A | eRNA |
| PLOD2 | eRNA |
| PAM | eRNA |
| PTMA | eRNA |
| YWHAQ | eRNA |
| C1S | eRNA |
| COL1A2 | eRNA |
| PSAP | eRNA |
| DAD1 | eRNA |
| PXN | target |
| RNF34 | target |
| RPLP0 | target |
| RAE1 | target |
| VAPB | target |
| COX6B1 | target |
| LIN37 | target |
| TMEM147 | target |
| ZNF146 | target |
| ASAP2 | target |
| CHD4 | target |
| VAMP1 | target |
| ZNF384 | target |
| PABPN1 | target |
| HALLMARK_EPITHELIAL_MESENCHYMAL_TRANSITION | pathway |
| HALLMARK_MTORC1_SIGNALING | pathway |
| HALLMARK_MYC_TARGETS_V1 | pathway |
| HALLMARK_OXIDATIVE_PHOSPHORYLATION | pathway |
| HALLMARK_DNA_REPAIR | pathway |
| HALLMARK_INFLAMMATORY_RESPONSE | pathway |
| HALLMARK_PROTEIN_SECRETION | pathway |
| HALLMARK_UNFOLDED_PROTEIN_RESPONSE | pathway |
| HALLMARK_TGF_BETA_SIGNALING | pathway |
| T cell CD4+ memory resting | immune cell |
| NK cell resting | immune cell |
| NK cell activated | immune cell |
| Macrophage M0 | immune cell |
| Mast cell resting | immune cell |
| aDCs | immune gene sets |
| CCR | immune gene sets |
| Check-point | immune gene sets |
| Macrophages | immune gene sets |
| MHC_class_I | immune gene sets |
| T_cell_co-stimulation | immune gene sets |
| Type_II_IFN_Reponse | immune gene sets |
